# Developing Pre-Service Teachers’ Capacity for Lesson Planning with the Support of Curriculum Resources

**DOI:** 10.1007/s11858-025-01685-0

**Published:** 2025-05-08

**Authors:** Birgit Pepin, Iveta Kohanová, Magdalini Lada

**Affiliations:** 1 Norwegian University of Science and Technology, Trondheim, Norway; 2 Eindhoven University of Technology, Eindhoven, The Netherlands

**Keywords:** Curriculum resources, Lesson planning, Pre-service mathematics teachers, Reasoning and proving, Textbook tasks

## Abstract

This study investigates how pre-service mathematics teachers (PMTs) develop the capacity to plan mathematics lessons regarding reasoning and proving (R&P) at a time of curriculum change in Norway. Lesson planning poses challenges for both pre-service and experienced mathematics teachers. In this EU-funded intervention study, PMTs were led through iterative cycles of learning about different approaches to R&P; designing and refining R&P tasks from reform-oriented textbooks and other resources; developing lesson plans; implementing lessons in the classroom; reflecting on and revising their plans; engaging in peer discussions. The goal was to develop Grade 8 lesson plans that supported pupil reasoning. Using a case study approach, data were collected through multiple methods, including pre- and post-intervention interviews with PMTs; reflective texts; lesson plan iterations (several versions throughout the intervention); and observation field notes. Results from the Norwegian data showed that providing PMTs with commonly used reform-oriented textbooks or teacher guides is insufficient. Instead, PMTs require additional support in two key areas: (a) Understanding different types of R&P across various mathematical domains and tasks, and (b) Developing comprehensive lesson plans that address all key components, such as learning objectives, activities, assessment, pupil thinking and misconceptions, and the teacher’s role. By framing curriculum design as part of teachers’ lifelong professional learning, this study contributes to ongoing research on how PMTs develop curricular expertise with the support of curriculum resources.

## Introduction

Referring to the topic of the Special Issue (Mathematics Textbooks for Curriculum Development and Instructional Reform), we investigate in this study which resources, and in which ways, were useful for pre-service- mathematics teachers (PMTs) for their lesson planning in terms of reasoning and proving (R&P), particularly at a time of curriculum change in Norway. We regard lesson planning as a form of curriculum development here. The research goal aims to bridge the theoretical underpinnings of curriculum resources with practical applications in lesson planning, emphasizing the role of curriculum resources (e.g., textbooks) as tools for instructional development.

In the context of curriculum reform, lesson planning is typically deeply connected to curriculum resources (e.g., textbooks) and their use, serving often as tools for operationalizing curricular changes. For example, textbooks (or other curriculum resources) often serve as a primary resource for translating curricular goals into actionable lesson plans (and finally classroom practices). Understanding this connection requires an analysis of how teachers interact with curriculum resources (e.g., textbooks) to design lessons that align with ‘new’ curricular objectives.

Lesson planning - particularly the ability to design mathematics lessons thoughtfully - is a crucial skill for all teachers, especially for PMTs. However, despite its significance, there remains a substantial need for research on factors influencing lesson planning and a shared understanding of how to develop teachers’ competence in this area. Key questions include what opportunities and challenges PMTs should encounter during teacher education, what types of lesson-planning experiences best support their growth, and what conditions and resources are necessary for effective development. Addressing these gaps can help to ensure that PMTs receive the structured support they need to develop strong lesson-planning skills.

Moreover, teachers - particularly PMTs - often struggle with lesson planning, especially when designing lessons ‘from scratch’ (Cevikbas et al., 2023). Research suggests that PMTs perform better when using templates to synthesize or adapt existing lesson plans (Lim et al., 2018). Additionally, Cevikbas et al. (2023) found that PMTs often face difficulties in selecting appropriate activities and problems for students, highlighting the need for greater support in this aspect of lesson planning.

Lesson planning becomes even more challenging during periods of curriculum change, particularly when key resources, such as textbooks, are being adapted. This paper reports on an EU-funded intervention study designed to better prepare teachers to teach R&P during a national curriculum reform in Norway. As part of the intervention, PMTs engaged in iterative cycles that involved exploring different approaches to R&P, designing and refining R&P tasks from reform-oriented textbooks, developing lesson plans, implementing them in the classroom, reflecting on and revising their plans, and engaging in peer discussion. Through this process, they worked to develop Grade 8 lesson plans that effectively fostered pupils’ mathematical reasoning.

As an initial clarification, we define curriculum here as the ‘path for learning’ and view curriculum work as the process of engaging with curriculum resources and designing for learning. In previous research (e.g., Pepin & Gueudet, 2018), we have defined mathematics curriculum resources as “all the material resources that are developed and used by teachers and students in their interaction with mathematics in/for teaching and learning, inside and outside the classroom” (p. 172/173).

The aim of this study is to explore and develop the curricular/lesson design skills demonstrated by Norwegian PMTs as they engage with curriculum resources during a period of curricular reform. We assume that PMTs can enhance their capacities to create effective lesson plans as they become more familiar with pupils and the existing curriculum over time. To investigate this, we pose the following research question: *How can pre-service mathematics teachers develop capacity for lesson planning regarding reasoning and proving at a time of curriculum change?*

Following this introduction, we present the theoretical frameworks and background literature relevant to this area of research. Next, we explain the methods employed in the study. We then present the results, followed by a discussion of the findings and our conclusions.

## Theoretical Frames and Related Literature

The theoretical frames and related literature for this study encompass five main areas: (1) curriculum; (2) teacher curriculum design capacity; (3) lesson planning; (4) reasoning and proving; and (5) curriculum resources as mediators between policy and practice.

### Curriculum

Curriculum encompasses the planned and organized framework for guiding learning experiences in educational settings. It includes not only the content or subjects to be taught but also the pedagogical strategies, assessments, and intended learning outcomes that shape the educational process.

Typically, the literature (e.g., Thijs & van den Akker, 2009) distinguishes between different curriculum representations. Usually, there are three levels (i.e. the intended, the implemented/enacted, and the attained curriculum), which can be split up in further two each, to make up six forms (Table 1).

**Table 1:** Forms of curriculum (Thijs & van den Akker, 2009, p. 10)

INTENDED	Ideal	Vision (rationale or basic philosophy underlying a curriculum)
	Formal/written	Intentions as specified in curriculum documents and/or materials (e.g., textbooks)
IMPLEMENTED/ENACTED	Perceived	Curriculum as interpreted by its users (especially teachers)
	Operational	Actual process of teaching and learning (also: curriculum-in-action)
ATTAINED	Experiential	Learning experiences as perceived by learners
	Learnt	Resulting learning outcomes of learners

The division into six representations, building on the work of Goodlad (1979), is particularly useful for the analysis of the processes and outcomes of curriculum innovations. For our study, we focused primarily on the ‘intended’ level, as our main concern was lesson planning. However, one of the lesson plans was also enacted and subsequently revised based on experiences during implementation and discussions afterwards.

### Teacher Curriculum Design Capacity

There are varied perspectives on PMTs’ curriculum design capacity (CDC) and lesson planning in teacher education, with no consensus on how CDC develops. PMTs are generally expected to acquire curriculum design during their training, often through lesson planning for teaching practice. However, mathematics education courses traditionally emphasize the transfer of mathematical content into teaching strategies, and PMTs often struggle to apply their knowledge in practice due to differing views on their role in curriculum design.

Remillard (2005) identifies four teacher roles in relation to curriculum: (a) conduit for curriculum, (b) meaning maker, (c) co-planner, and (d) curriculum leader. While the “conduit” role involves strictly following the curriculum, the other roles position teachers as active designers or collaborators, supporting reform-oriented practices that expand instructional strategies and refine teachers’ beliefs.

Teaching can be viewed as a design process, with lesson plans serving as tangible evidence of this activity. Several studies (e.g., Brown, 2009; Pepin et al., 2017) highlight teachers as designers who adapt resources, navigate constraints, and develop strategies to achieve instructional goals. This process is shaped by both the teachers’ competencies and their interactions with curriculum materials, aligning with frameworks such as the Documentational Approach to Didactics (Gueudet et al., 2012). Effective curriculum design requires deliberate decision-making, including goal setting, understanding student needs, selecting appropriate resources, and planning assessments. Teacher design capacity is inherently goal-oriented, requiring clear guiding principles, adaptability, and reflective practices throughout both the planning and enactment phases (Pepin, 2018).

### Lesson Planning

Lesson planning skills are essential for teachers to perform their daily work efficiently and effectively. For PMTs, selecting and adapting appropriate mathematical tasks (e.g., from textbooks) and designing instructional units or individual lessons are critical components for their professional development (Sadak, 2021). A well-developed and thoughtful lesson plan serves as a roadmap, ensuring that all necessary content is covered in an organized manner. Effective lesson planning is widely regarded as crucial for successful teaching and learning, helping teachers communicate clearly, manage time efficiently, engages students, and conduct accurate assessments and evaluations.

However, for PMTs, the challenge of deciding what to teach is often compounded by difficulties in determining how to present the material. They frequently express a need for specific curriculum resources to guide their decisions. Adapting content to meet diverse learning needs is a complex task (Fantilli & McDougall, 2009), requiring PMTs to navigate fundamental pedagogical questions while simultaneously familiarizing themselves with the curriculum. Lim et al. (2018) found that PMTs struggle to identify the mathematical ideas within a given task and translate them into a coherent plan. Similarly, Chen and Zhang (2019) reported that PMTs often lack the knowledge required to modify lesson plans to meet the needs of diverse student groups. Their findings suggest that PMTs tend to adopt a rigid, formulaic teaching style with little didactic flexibility.

A review study by Cevikbas et al. (2023) synthesized key findings from existing research, categorizing challenges related to lesson planning into four major themes: (1) PMTs’ dispositions and their influence on developing and implementing lesson plans, (2) quality aspects of lesson plans, (3) difficulties in lesson planning, and (4) the relationship between these factors and broader teaching competencies.

### Reasoning and Proving

In recent years, several European countries have revised their national mathematics curricula, particularly with regard to R&P (Schmidt et al., 2022). Policy documents and national standards emphasize the importance of teaching mathematics with understanding – focussing not only on procedures and theorems but also on the reasoning behind them, proving results in algebra and geometry, justifying mathematical thinking, and constructing valid arguments (NCTM, 2009). The emphasis on R&P stems from the idea that these processes are fundamental to the construction and validation of mathematical knowledge, making them essential for learning and understanding mathematics (Weingarden & Buchbinder, 2023). Hanna and Barbeau (2008) propose a set of principles to guide mathematics teachers in integrating R&P in their instruction: “(1) Reasoning and proving must be fully embedded in the existing mathematics curriculum; (2) Emphasis should be put on deductive reasoning in knowledge production and validation, while (3) Using language, notation, and representations within the conceptual reach of the students.” (p. 2).

A review of research on R&P over the past decades by Stylianides and Stylianides (2017) highlights extensive knowledge on students’ difficulties with proof and the challenges teachers face in teaching it. They call for further research and the development of strategies to support mathematics teachers in engaging students with R&P and designing lessons that foster this engagement. They particularly stress the need to better prepare teachers to enact R&P-focussed instruction, suggesting that targeted interventions could help PMTs develop expertise in this area.

Various frameworks have been proposed to introduce PMTs to proof and its teaching in a structured way. Stylianides and Stylianides (2022) suggest a coherent approach based on two multi-year design experiments, outlining a learning trajectory with two key milestones: (1) recognising the need to learn about proof and (2) ‘developing an operationally functional conceptualization of proof.’ Similarly, Buchbinder and McCrone (2020) propose the *Mathematical Knowledge for Teaching Proof* framework, which defines the knowledge, dispositions, and practices teachers need to teach mathematics through R&P. This framework is rooted in socio-cultural and situated perspectives on teacher learning (Borko et al., 2000), which view knowledge and learning as situated in physical and social contexts and developed through active participation in social practices.

Despite the importance of R&P in mathematics education, little is known about how future teachers become proficient in integrating R&P into their instructional practices. While R&P is acknowledged as central to student engagement with mathematics, there have been few efforts to develop structured learning trajectories or experiences for PMTs in teacher education. One notable exception is the study by Weingarden and Buchbinder (2023), who draw on commognitive theory to characterize this aspect of PMTs’ professional learning. Their research examines PMTs’ pedagogical discourse through lesson plans they designed, enacted, and modified in a university course, arguing that lesson plans serve as both proxies for teaching practices and valuable tools for understanding PMTs’ professional learning.

### Curriculum Resources as Mediators Between Policy and Practice

In an earlier study, Pepin et al. (2013) investigated mathematics curriculum documents, commonly used textbooks and teacher ‘curricular practice’ in relation to educational traditions in France and Norway. One of the study’s key aims was to develop a deeper understanding of the connections between policy, textbooks and teacher curricular practices in mathematics. Results showed that while both French and Norwegian curricular documents and practices were shaped by egalitarian values, these values were interpreted and ‘lived’ differently in each country. The study also argued that mathematics textbooks serve as a crucial interface between culture, policy and curricular practice, functioning as a pivotal resource in teachers’ curricular decision-making.

Large-scale international studies on mathematics student achievement, such as “Trends in International Mathematics and Science Study (TIMSS)” have suggested that textbooks historically played a central role as the primary curriculum resource, shaping the provision of educational opportunities (Haggarty & Pepin, 2002; Rezat, 2012). Valverde et al. (2002) contend that

“Textbooks are commonly charged precisely with the role of translating policy into pedagogy. They represent an interpretation of policy in terms of concrete actions of teaching and learning. Textbooks are the print resources most consistently used by teachers and their students in the course of their joint work.” (p. viii)

However, with the advancement of digital curriculum resources – including e-textbooks and education platforms – the role of traditional textbooks has evolved. More recently, AI-driven resources (e.g., ChatGPT) have introduced new opportunities for both teachers and learners, gradually transforming the education landscape, including mathematics education (e.g., Pepin et al., 2024). While these developments are significant, they were not the focus of the present study.

## Methods

In terms of general features of the intervention study, its aim was to better prepare teachers for the challenges of teaching R&P, particularly during curricular reform. As part of the European Union-funded MaTeK project (Slavíčková et al., 2022), we developed and systematically studied an intervention to address this issue.

The intervention was implemented with minor variations at five partner institutions (in the Czech Republic, Italy, Norway, Slovakia, and Turkey) during the 2022/2023 academic year. It guided PMTs through cycles of: (1) Learning about different approaches to R&P; (2) Preparing mathematical R&P tasks and lesson plans (as part of a learning trajectory) for teaching in secondary classrooms in their respective countries, integrating R&P aspects with the curriculum; (3) Reflecting on the lesson plans produced by different groups; (4) Implementing one of the commonly agreed -upon and selected lesson plans in a secondary classroom; and (5) Reflecting on the implemented lesson to refine the lesson plans; (6) Presenting and discussing the final lesson plans at an international meeting; and (7) Developing new lesson plans in internationally mixed groups. However, this article focuses solely on the Norwegian case, detailing the context, participants, content, data collection strategies, and analyses.

### Context of Norway

The Norwegian education system consists of a seven-year primary school (grades 1–7), a three-year lower secondary school (grades 8–10), and a three-year upper secondary school (grades 11–13). The curriculum emphasises providing all pupils with the same content and competencies, fostering a holistic and egalitarian learning environment.

In 2017, teacher education in Norway transitioned to a 5-year integrated master’s, known as ‘Master i grunnskolelærerutdanning’ (MGLU 1-7 or MGLU 5-10). Students in the MGLU 5-10 programme must choose between mathematics or Norwegian as their core subject in their first three years of teacher education, whereas MGLU 1-7 students do not have this requirement. Additionally, all teacher education students must select a subject (e.g., mathematics) as their specialisation for their master’s studies.

Over the past 50 years, Norway has undergone six curriculum changes, significantly modifying the mathematics curriculum (Borge et al., 2022). The most recent are the 2006 curriculum (LK06) and the 2020 curriculum (LK20), with LK20 considered a ‘renewal’ rather than a reform of LK06.

LK06 was the first purely competency-based curriculum, inspired by the PISA framework (OECD, 2003). It organized competence aims across several grade bands (1–4, 5–7, 8–10). and included mathematical reasoning as part of its broader focus on mathematical competence. LK06 emphasised problem-solving and modelling to analyse and validate solutions, alongside communicating and reasoning about mathematical ideas. It also highlighted understanding and using symbolic language to interpret various texts and engage in logical reasoning. However, explicit references to reasoning were limited, and terms like ‘proving’ or ‘proof’ were not mentioned.

In LK20, mathematics competence aims for grades 1-10 are organised by grade, with 10 to 13 aims per year. Each aim includes a verb denoting a competence and a noun clause outlining a specific mathematical domain, such as “explore and argue for formulas for area and volume of three-dimensional figures” (Norwegian Directorate for Education and Training, 2019, p. 14). The verbs used range from ‘calculate’, ‘carry out’, ‘represent’, and ‘use’, to ‘explore’, ‘formulate’, ‘generalize’, ‘explain’, and ‘argue’.

LK20 also introduced six process-oriented core elements, emphasizing how pupils engage with mathematical concepts: *exploration and problem solving*; *modelling and applications*; *reasoning and argumentation*; *representation and communication*; *abstraction and generalization*; and *mathematical fields of knowledge* (including numbers, algebra, functions, geometry, statistics, and probability).

The core element ‘reasoning and argumentation’ applies broadly across all topics and grades, without explicit reference to formal or axiomatic approaches. Reid (2022), in an analysis of LK20 for grades 1–10, described reasoning as encompassing various aspects:

“The process of reasoning involves following, assessing and understanding mathematical chains of thought; it is related to the grounds of mathematics; the product of reasoning can be formulated; and giving grounds for that product is central to argumentation” (p. 302).

The verb *prove* appears only once in LK20, under *reasoning and argumentation*: “Argumentation in mathematics means that pupils give reasons for their approaches, reasonings, and solutions, and prove that these are valid” (Norwegian Directorate for Education and Training, 2019, p. 3).

However, it is not associated with formal proof nor linked to a specific mathematical topic. Processes related to mathematical reasoning, as defined by Jeannotte and Kieran (2017), extend beyond ‘reasoning and argumentation’ to the other core elements, emphasizing exploring patterns, identifying relationships, employing various strategies, communicating reasoning, and focusing on understanding rather than merely finding solutions.

### Participants

The study participants were enrolled in the MGLU 5-10 programme, specializing in mathematics. The intervention was conducted as part of the *Learning and Teaching of Mathematics* (LTM) course in PMTs’ 4th year of study. In total, 42 PMTs took the course, but only 21 agreed to participate in the study. Out of these, five did not participate in three intervention sessions, thus they were excluded from the study after all the data was collected. Therefore, the final sample consists of 16 PMTs (10 females, 6 males).

During their first three years in the MGLU 5-10 programme, PMTs take mathematics education courses focused on developing pedagogical content knowledge for teaching school mathematics, rather than ‘pure’ mathematics. These courses encourage alignment with current research and the mathematics curriculum, using exploratory, experimental, problem-solving methods, and investigative activities.

Regarding R&P, the courses involve generalizations, conjecturing, argumentation, justifications, and proving topics like figural patterns, odd/even numbers, prime numbers, and geometric measurements. PMTs encounter formal proofs and logic to a limited extent in a second-year course on school geometry, which includes Euclidean geometry and its axiomatic system.

Before the intervention, PMTs had completed 95 school-based practice days over four years, including five days of observational practice. Although they specialized in mathematics, they did not always teach it during their practice, depending on the specialization of their mentor teacher. PMTs worked in mixed groups and collaboratively planned and taught lessons, sometimes independently. Lesson planning, primarily covered in pedagogy courses, follows the ‘Didactical Relationship Model’ (Hiim & Hippe, 2006), widespread in Scandinavian Didactics, emphasizing content, learning activities and methods, and justification. Plans are reviewed with mentors and occasionally with visiting teacher educators.

### Intervention

The intervention consisted of four 180-minute long sessions in which the PMTs, working in groups and guided by the first two authors, engaged in iterative cycles to develop Grade 8 lesson plans that supported pupils’ reasoning. PMTs were free to choose the mathematical topic, with only two requirements: it had to be relevant to the new curriculum and use the “curriculum spider web” template (van den Akker, 2003).

**Fig. 1:**
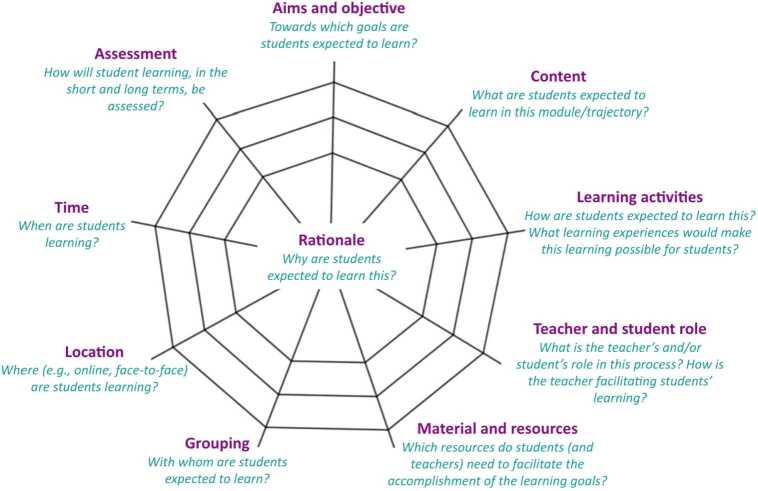
Curriculum spider web (van den Akker, 2003)

Van den Akker’s (2003) ‘curriculum spider web’ (Fig. 1) highlights the key facets of curriculum development, including aims, content, learning activities, the teacher’s role, resources, and assessment. The metaphor of a ‘spider web’ is particularly useful, as these facets are intricately interwoven and interconnected. At the same time, a spider web is also fragile - if pulled too strongly in one direction, it can break.

One of the PMTs’ lesson plans was selected and enacted in an 8th-grade classroom by an in-service teacher, who followed the designed lesson plan. This lesson was video-recorded and later analysed in the fourth intervention session, with discussions focussing on teacher moves, noticing of pupils’ learning, and identifying necessary modifications to the lesson plan. The first three intervention sessions included the following themes:

- Learning about different modes of reasoning (Silverman & Even, 2015), designing or redesigning R&P tasks found in commonly used reform-oriented textbooks and other resources;
- Teacher moves supporting student reasoning (Ellis et al., 2019);
- The curriculum ‘spider web’ (van den Akker, 2003) as a framework for curriculum development and lesson planning;
- Discussing the first version of lesson plans with peers and teacher educators, followed by reflection and redesign;
- Socio-mathematical norms (Stephan, 2020).

**Fig. 2:**
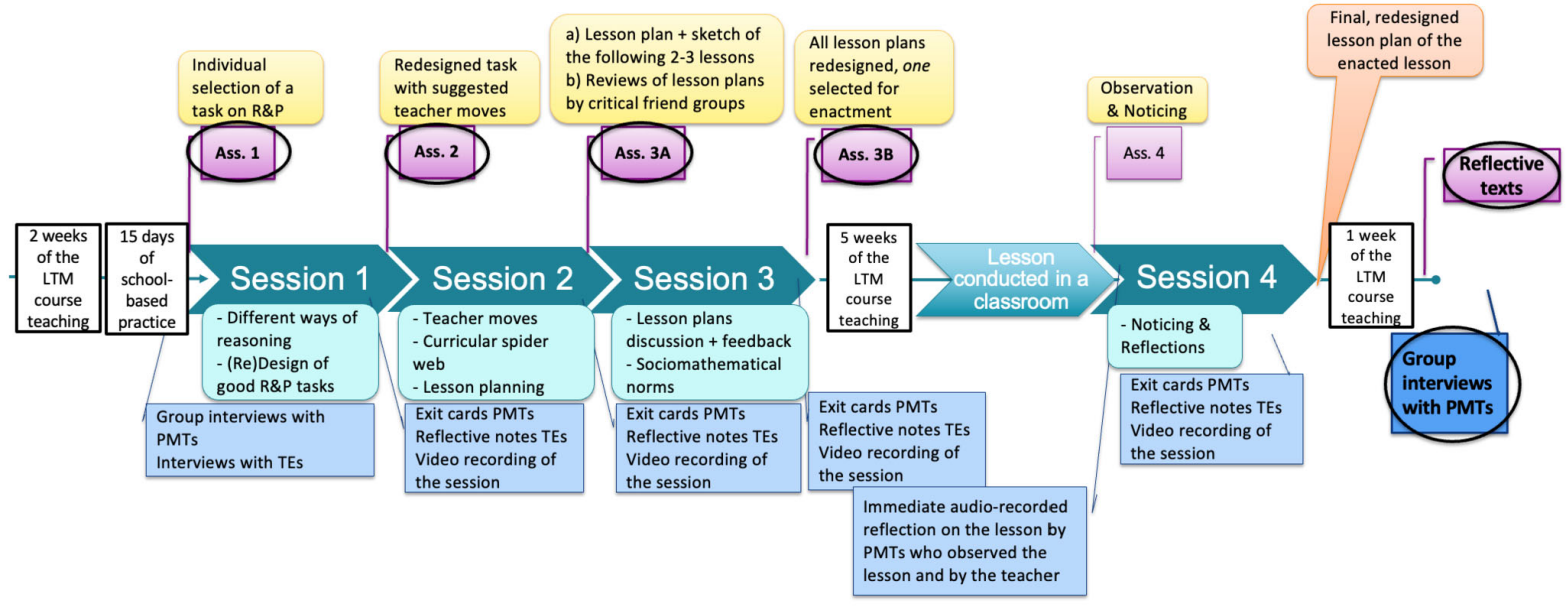
Timeline of the intervention with data sources collected in each stage (analysed sources are highlighted by circles)

An overview of the intervention, including various data sources, is provided in Fig. 2.

### Data Collection Strategies

Before each session, PMTs were assigned selected readings (listed above), which were then discussed in class. Additionally, they completed assignments related to the theme of each session. After Session 1, all 42 PMTs in the LTM course worked in nine groups of four to five, with the 16 participants in this study working in groups 1, 2, 4, and 5. Table 2 summarizes the assignment content analysed. Following the intervention, participants wrote reflective texts on their learning and took part in post-intervention group interviews. Since the reflective texts were not mandatory, only ten PMTs submitted them; these participants are referred to as S1, S2, …, S10. Of these, seven PMTs were also available for interviews, which were conducted in three group interviews (with 3, 2 and 2 participants respectively).

**Table 2:** An overview of data collection strategies

Data source	Individual [I] or Group [G] (number)	Content	Analysis method
Assignment 1	I (16)	Selection/design of an R&P task, justification of the task, identification of type(s) of reasoning required in the task	Qualitative analyses of changes in PMTs’ tasks and lesson plans with connections to specific intervention activities or design principles
Assignment 2	G (4)	Selection/design of a new R&P task or redesign of the Ass. 1 task, suggestion of possible teacher moves for this task	
Assignment 3A	G (4)	Design of lesson plan around task from Ass. 2 + sketch for subsequent 2-3 lessons; written review of lesson plans among critical friend groups	
Assignment 3B	G (4)	Redesigned lesson plans based on the received feedback in session 3	
Reflective text (r)	I (10)	Learning outcome, Surprises, Benefits, Group collaboration, Lesson planning confidence, R&P support confidence, Areas for improvement in R&P	Qualitative open-coding of PMTs’ self-reported Evidence of learning from the intervention (types of knowledge/practices) with connections to specific intervention activities or design principles
Post-interviews (i)	G (3)	Lesson planning, R&P, learning from observing the enacted lesson, Intervention’s key learning	

### Data Analysis

The data analysis followed two distinct approaches:

#### Intervention Analysis

A holistic review of PMTs’ learning experiences was conducted by triangulating assignments, reflective texts, and interviews. The reflective texts and post-interview transcripts were analysed to identify recurring themes and insights into PMTs’ learning experiences and challenges. The analytical approach was both inductive (as we had no predefined set of learning experiences) and deductive (as our analysis was strongly influenced by the literature described in the theoretical background).

#### Curriculum and Textbook Analysis

To contextualize findings, we analysed national curriculum developments (LK06 and LK20) with a focus on R&P, applying Hemmi et al.’s (2013) framework and methods similar to those used by Valenta and Enge (2020). Verbs and related noun clauses in competence aims were categorized into six proof-related categories: [Proof], [Argumentation], [Investigations], [Structure], [Definitions], [Logic], as well as a seventh category, [non R&P], along with the associated mathematical topics. Table 3 illustrates changes in aims and verb categorization between LK06 and LK20. This study focuses exclusively on lower secondary competence aims (grades 8–10), as all PMTs’ lesson plans pertain to this level of the Norwegian school system.

**Table 3:** Examples of competency aims from LK06 and LK20 and categorization of verbs

LK06	LK20
use *[non R&P]* factors, powers, square roots and prime numbers in calculations	use *[non R&P]* powers and square roots in exploration and problem solving, and argue *[Argumentation]* for approaches and results
estimate *[non R&P]* and calculate *[non R&P]* length, circumference, angle, area, surface, volume, speed and density, and use *[non R&P]* and change *[non R&P]* scales	explore *[Investigations]* and argue *[Argumentation]* for formulas for area and volume of three- dimensional figures

Since topic of ‘patterns’ appeared in all students’ lesson plans, we focused our textbook analysis on this topic and its related competence aims. In LK20, a competence aim related to pattern generalization was rephrased, split, and moved from grades 5–7 in LK06 to grade 8 (“Describe and generalize patterns in one’s words and algebraically”) and grade 9 (“Describe, explain and present structures and progressions in geometric and numerical patterns”).

Our textbook analysis examined how two commonly used textbook series - *Matematikk 8* and *Matematikk 9* (Hjardar & Pedersen, 2020a,b) and *Matemagisk 8* and *Matemagisk 9* (Kongsnes & Wallace, 2020a,b) - address these aims. These textbooks, revised to align with LK20, were also used by our PMTs in their lesson plans.

First, we identified the types of patterns in solved examples, explanatory texts, and unsolved tasks. Most patterns involved numerical or figural sequences and patterns of natural number properties. As these were also the predominant types of patterns in our PMTs’ lesson plans (see Table 4), we focussed our analysis on these.

**Table 4:**
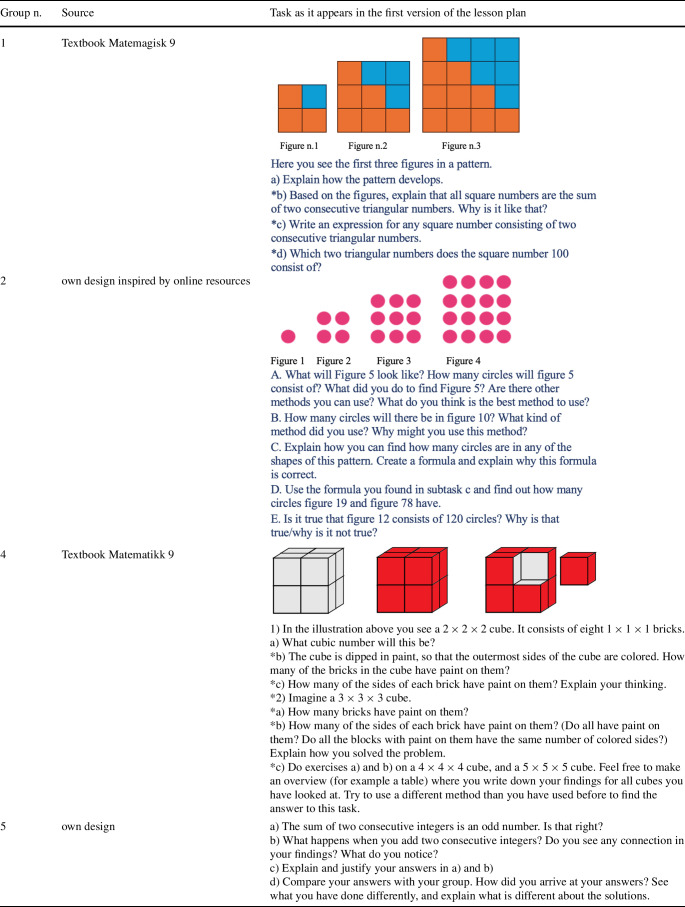
Overview of tasks around which PMTs designed their lesson plans, * indicates amendment of an original textbook task

Second, we examined the reasoning processes associated with pattern generalization. Guided by Radford’s (2006) definition, we analysed each of the three stages of algebraic generalization of patterns and further categorized

- the type of identification of a pattern as either recursive or explicit,
- the mode of generalization as either numerical or figural (Rivera & Becker, 2005), and
- the type of expression as either factual, contextual or symbolic (Radford, 2006).

We coded the different stages of an unsolved task based on the form of the questions. For example,

- “How many more bricks are in figure no. 11 than in figure no. 10?” (Kongsnes & Wallace, 2020a, p. 118) was coded for identifying the recursive rule.
- “What is the connection between the total number of columns of the figure and the number of the figure?” (ibid, p. 116) was coded as explicit and figural generalization, as it focusses on the connection between the figure number and the total number of elements.
- “Explain with words how figure n. 50 looks like” (ibid, p. 118) was coded as factual.
- “Explain with words how you find the total number of matches in a specific figure” (ibid, p. 193) were coded as contextual.

Third, we searched for reasoning processes related to validating (Jeannotte & Kieran, 2017). While no validating processes were found in solved examples or explanatory texts, we identified unsolved task questions that invited pupils to provide an argument. Where possible, we coded the type of argument as either a *generic example* or a *demonstration*.

### Reliability and Validity

In terms of reliability (referring to the consistency and dependability of the findings), we triangulated our data sources to cross-check and corroborate insights across different perspectives. Our approach included: (1) inter-coder agreement and reliability – we established consistency in coding (e.g., for lesson plans, reflective texts, and interviews) through discussions and alignment of coding-framework (e.g., textbook analysis). (2) Transparent research documentation – we maintained a detailed log of all research steps (see Fig. 2), ensuring that the study could be replicated. (3) Standardised protocols – whenever possible, we followed standardised guidelines for data collection and analysis, such as structured data collection protocol, a rubric for lesson plan analysis of lesson, and a consistent procedure for conducting interviews and analysing curriculum documents.

In terms of validity (to ensure that the research accurately reflects the phenomena being studied), we addressed four aspects of validity: (1) Construct validity- we clearly defined key concepts (e.g., curriculum) to ensure alignment between data collection strategies and theoretical constructs (e.g., reflective texts to capture participants’ views); (2) Content validity – we ensured that our instruments (e.g., interview questions) comprehensively covered all aspects of the lesson planning; (3) Internal validity – we identified potential biases (e.g., preconceived notions about curricular reform) and cross-checked findings from lesson plans with interview and reflective text data to ensure consistency; (4) External validity – we clarified the study’s context (see Context of Norway) to assess how the findings might generalize to other educational settings.

Despite these efforts in terms of reliability and validity, the study’s generalisability is limited, partly due to the small number of participating PMTs. However, we maintain that our rigorous approach ensures a high degree of validity, allowing us to offer meaningful new insights.

## Results

In this section, we present the results of our analyses of: (1) National curriculum development; (2) Commonly used textbooks; (3) Lesson plans designed and revised by PMTs; and (4) Reflective texts and post-intervention interviews.

### Analysis of the Curriculum Changes with Respect to R&P

The analysis of LK06 and LK20 revealed an increase in the proportion of verbs related to R&P in competence aims:

• *Argumentation*: about 6% in LK06 vs. about 21% in LK20,

• *Investigations*: about 18% in LK06 vs. about 28% in LK20.

This increase led to a decrease in non-proof-related verbs in competence aims, dropping from 67% in LK06 to 45% in LK20. The proportion of verbs in the category *Structure* remained stable at 5% in both curricula. The *Logic* category showed a decrease from 1.3% in LK06 to 0% in LK20, while the *Proof* category remained at 0% in both.

Topic-specific analysis showed notable increases in R&P – related competence aims:

- Numbers and Algebra: from 10% in LK06 to 19.4% in LK20.
- Functions: from 3.7% in LK06 to 14.9% in LK20.
- Smaller increases were observed in Statistics and Probability and Geometry and Measurement.

These findings indicate that LK20 places a stronger emphasis on R&P across all topics, with particularly pronounced growth in algebra and functions.

In summary, LK20 for grades 8-10 places greater emphasis on investigations and argumentation (including conjecturing and justification) than LK06, across all mathematical topics. However, both curricula place little focus on definitions, logical thinking, and deductive reasoning, aligning with Valenta and Enge’s (2020) conclusions for LK20 as a whole. As a result, the development of pupils’ competence in proof and proof-related work is largely dependent on teachers and textbook authors. Given the broad competence aims and the integration of reasoning processes into multiple core elements, teachers’ interpretations of R&P may vary, leading to potential challenges.

### Textbook Analysis

As mentioned earlier, our analysis examines two textbook series, focusing on the types of patterns presented, the approach to generalization, and the extent to which reasoning processes related to validation are encouraged.

**Fig. 3:**
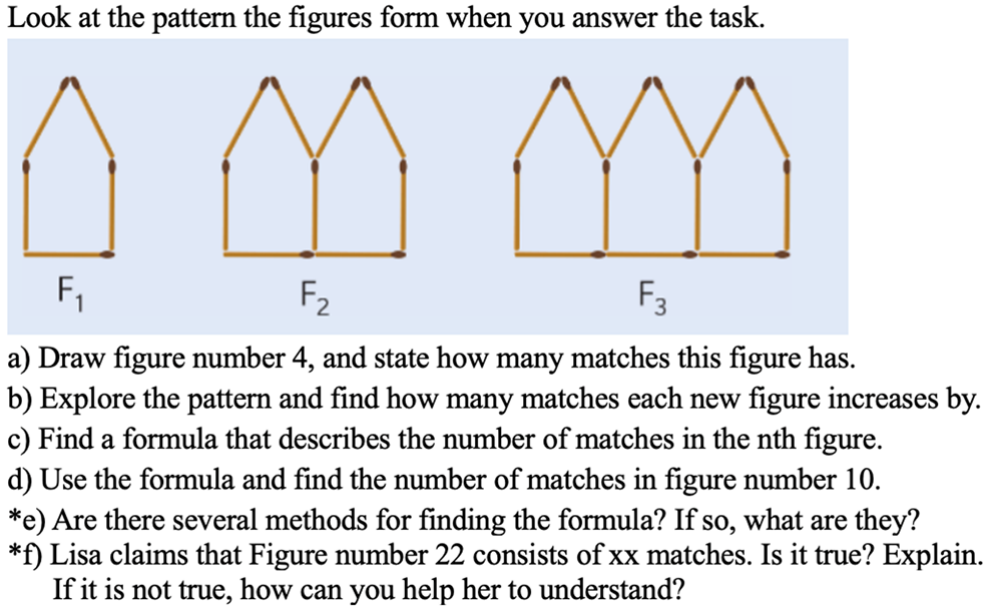
Group 5’ extension (indicated with a *) of a textbook task used in their lesson series

In Matematikk grade 8, most patterns are numerical sequences, whereas the grade 9 textbook features only figural sequences. In both grades, generalization begins with (a) pattern identification, mostly recursive, followed by (b) algebraic generalization leading to symbolic expressions. Only one question was coded as inviting for a contextual generalization. Occasionally the symbolic expression is then used to find a distant term in the sequence. This reasoning path is illustrated in the example found in the grade 9 textbook, which is the task that group 5 included modified in their revised lesson plan (see Fig. 3). It is noteworthy that processes related to validating are almost absent in this textbook with only one question prompting pupils to argue for a claim.

Matemagisk, in both grades, offers a wider variety of figural sequences, which become gradually more complex. The grade 8 textbook introduces different strategies and includes a mix of all types of identifications, generalizations and expressions. While no single reasoning path dominates, there is a clear preference for figural generalization, reflected in tasks prompting pupils to describe visual patterns and mark different parts of figures with distinct colours. Symbolic expressions are frequently preceded by factual or contextual ones. The grade 9 textbook emphasizes decomposing complex figures into simpler ones, investigating part-whole relationships, and using symbolic expressions. An example is the task chosen by group 1 (see Table 4). Additionally, the grade 9 textbook includes tasks based on patterns from natural number properties, which explicitly invite validation processes, particularly through generic arguments.

To summarize, the two textbooks differ in their approach to generalizing patterns, illustrating the effect of broadly formulated competence aims and the incorporation of R&P processes across different core elements. For example, since the grade 8 competence aim refers to “patterns” without specification, the textbooks focus on different types of patterns. Additionally, the opportunities for engaging with R&P differ: Matematikk emphasises reasoning processes related to explorations and generalization, Matemagisk incorporates validation processes.

### Lesson Plans

The starting point for the lesson plans was a task that PMTs either designed themselves or selected from textbooks or other curricular resources as part of Assignment 1. These tasks were intended to support the reasoning of Grade 8 or 9 pupils. Of the 16 PMTs, eight submitted a figural pattern task, one a number pattern, six chose a geometry task, and one selected a combinatorial task. In Assignment 2, groups 1, 2, and 4 elaborated on a figural pattern task from Assignment 1, while group 5 designed a new task on whole number properties (Table 4). PMTs suggested possible teacher moves, and in Assignment 3A, they submitted the first version of a Grade 8 lesson plan built around these tasks, including sketches for two to three subsequent lessons. Group 5 designed a series of four lessons, combining their Assignment 2 task with figural pattern tasks from Assignment 1 (Matematikk 9 textbook, Fig. 3), an online resource, and a whole numbers task discussed in Session 2.

Since all four lesson plans focused on the generalization of figural patterns or whole numbers properties, PMTs aligned their revised lesson plans with the same LK20 competency aim for 8th grade: “describing and generalizing patterns in one’s own words and algebraically”. The rationale for the lesson (series) was rooted in the Norwegian National Curriculum, emphasizing core elements and importance of reasoning. For example, group 5 justified their approach by stating: “The fact that pupils are able to reason and potentially prove will be of great help for further learning, within all topics in mathematics”. The main learning activities were built around tasks from textbooks or other curricular resources (Table 4), and combined with several tasks planned to be used in the subsequent lessons. However, all groups modified or extended textbook tasks to enhance reasoning and justifications.

In terms of classroom organisation and the teacher’s role, the pupils mainly worked in pairs or small groups (3-4 pupils), alternating with whole-class discussions. A strong emphasis was placed on communication, encouraging pupils to share different strategies both among themselves and with the teacher. The teacher’s role was of a facilitator/guide, incorporating specific teacher moves (Ellis et al., 2019). For example, “the teacher should respond to pupils’ reasoning by encouraging them to correct mistakes and in the plenary discussion represent pupils thinking”.

Moreover, group 2 emphasised the importance of viewing failure as a learning resource, helping pupils address difficulties and misconceptions. In terms of assessment, assessment methods included both informal (e.g., teacher observations) and formative techniques (e.g., oral reviews of pupils’ strategies in plenary discussions). Common resources across all lesson plans included: (1) blackboard, stationery, notebook, and curriculum materials (e.g., prepared task sheets, homework sheets). Group 4 additionally listed stickers (“as an alternative to paint”), manipulatives or a 3D programme. In terms of lesson design and reasoning paths, the lessons were student-centred, aiming to develop descriptive and reasoning skills related to patterns. Across all groups, the path of reasoning followed a similar sequence: Identification of a pattern → near generalization → expression of the generality → application of the formula.

Comparing the initial and revised lesson plan versions, we observed:

- Clearly defined assessment methods in all groups’ final lesson plans.
- Refined tasks, placing greater emphasis on reasoning and justifications.
- More structured lesson sequences, with clearer learning goals, well-defined tasks, and a systematic progression ensuring pupils build on prior knowledge effectively.

However, most R&P – related improvements in the revised plans were driven by teacher educator feedback in Session 3. Peer feedback primarily addressed general pedagogical aspects, such as rewording learning goals to make them more accessible for pupils.

### Analysis of Reflective Texts and Post-Intervention Interviews

The reflective texts (r) and post-interviews (i) revealed three key areas where PMTs reported learning and adapting their knowledge and practices related to lesson planning.

#### Understanding R&P and Different Ways of Reasoning

Participants reported a deeper understanding of different types of reasoning and the *complexity of R&P* in school mathematics – an essential skill for adapting to new curriculum demands. PMTs realized that reasoning is not just about explaining steps but also about justifying why those steps are taken. As S9 reflected:

I’ve learned how big a difference it is between pupils telling what they’ve done and pointing to the correct answer versus being able to explain how they did what they did and why it is right. (r)

Similarly, S5 described how this enhanced understanding shifted his perspective on pupils’ learning:

It also really opened my eyes when we had the class about planning sessions, how much you put into it … […] when you’re in the classroom. [a teacher asks:] Yes, how did you come up with the answer? [a pupil answers:] I used that rule here, like that. [the teacher replies:] Yes, good. Then you have justified why you did something, but that doesn’t say anything about what they [pupils] can do. It’s just what they remember, so it’s kind of gotten a lot deeper, I started thinking a lot more about it. (i)

This enhanced understanding was also evident in how PMTs redesigned tasks from textbooks and other resources. For example, group 5 added two subtasks (Fig. 3) to an exercise from Matematikk 9 (Hjardar & Pedersen, 2020b) in their revised lesson plan series. These additions aimed to encourage multiple strategies and promote justification, aligning with the LK20 curriculum core elements related to R&P.

The understanding of the complexity of R&P also shaped PMTs’ beliefs about its importance in fostering understanding and problem-solving, even at the primary school level. For example, PMT S4 reflected: “I think I have become much more aware of the fact that reasoning and proof must be brought in, to create understanding” (i).

Regarding *different ways of reasoning*, some PMTs emphasised the importance of identifying these approaches and reflecting on their teaching practices. As S2 noted:

If all [pupils] refer to authority, then here is maybe something you need to change ... the ways you show. And therefore, it is important to be familiar with the different ways of reasoning. (i)

Some PMTs also considered these different ways of reasoning when thinking about pupil learning: “… how can I help pupils move from empirical reasoning to deductive reasoning?” (S5, r). However, several PMTs acknowledged their ongoing struggles with different ways of reasoning and expressed a desire for more knowledge and practical experience in this area.

#### The Role of the Teacher

All PMTs emphasised the crucial role of the teacher and specific teacher moves in supporting pupils’ reasoning. As S4 reflected:

Reasoning is not something that just comes as a by-product in a way, but it is something that as a teacher you have to fish for, or you have to sort of, you can’t just do a task and expect it to come, you have to kind of get it out of them ... Yes, you have to … ask them [pupils] the right questions, so that reasoning and proof will come. (i)

Furthermore, all PMTs agreed that teachers must cultivate a *classroom culture*, where justifying solutions becomes a natural part of learning. S2 highlighted this idea: “I think that a classroom culture for reasoning is a core element for reasoning in mathematics to become a natural part of the subject” (r).

#### Lesson Planning and Task Selection/Design

All PMTs acknowledged the importance of lesson planning, particularly when it came to critically evaluating textbook tasks and their potential to support R&P. For example, S7 reflected on whether tasks “actually require reasoning, or if reasoning can follow, under certain circumstances” (i).

Additionally, PMTs mentioned other components of a lesson plan. For instance, S10 reflected on pupils’ grouping: “when you let them sit in pairs to work, so you kind of have to share yourself a lot and you do not have enough time for each group” (i). Moreover, PMTs realised that teaching R&P can permeate all mathematical topic areas in the curriculum.

I also have to work further on how all the various topics in mathematics can function in a reasoning manner. With this, I think that in our teaching there was a lot of focus on figural numbers, and how to make this reasoning. But I still have to work on how reasoning can work in all the other topics we come across in teaching. In particular, I am interested in how to facilitate how to teach reasoning with programming. As programming has entered the new curriculum. (S4, r)

## Discussion of Results and Conclusions

In addressing the research question (*How can pre-service teachers develop the capacity for lesson planning regarding reasoning and proving during a time of curriculum change?*), we argue that PMTs require targeted support to develop their capacity for lesson planning, particularly in terms of carefully chosen resources and the integration of R&P, especially during periods of curriculum change. Simply providing commonly used reform-oriented textbooks or teacher guides is insufficient. PMTs need additional support in the following areas: (a) understanding the types of R&P in different mathematical areas and tasks, (b) how to elaborate comprehensive lesson plans that consider all lesson aspects (e.g., aims, activities, assessment, teacher role), and (c) understanding the curriculum changes and their implications for mathematics tasks and teaching. Our intervention addressed these needs through targeted sessions and specific resources introduced and used during the sessions (e.g., curriculum spider web; session/discussion on various ways of reasoning with examples).

However, curriculum changes presented additional challenges. Despite their experience (95 days of school placement), Norwegian PMTs still required support because: (1) general pedagogy courses appeared to be insufficient, and (2) their first three years of mathematics education courses (during the curriculum change) did not adequately support the conceptualization of R&P and its teaching under the reformed curriculum. Furthermore, as former students taught under the LK06 curriculum, the PMTs lacked direct experience with R&P teaching, as highlighted in interviews.

In terms of results, it was interesting to note that the PMTs’ selection of R&P tasks was largely based on what they found in commonly used textbooks, and what they had experienced in their teacher education (‘pattern spotting’). Working with R&P tasks helped them recognise how R&P permeates the curriculum and can be implemented across different areas, emphasising the importance of socio-mathematical norms in teacher education.

Regarding curriculum resources, we argue that careful consideration is needed, as they are crucial tools for structured lesson planning. Evidence showed that the following resources were key to PMTs’ development of lesson planning capacity: textbooks (evidence: choice of tasks, interviews); the National Curriculum (evidence: lesson plans); readings and sessions on modes of reasoning and teacher moves (evidence: reflective texts, interviews); the curriculum spider web (evidence: lesson plans, reflective texts); and human/social resources, such as collaborative work with peers and teachers (evidence: reflective texts, interviews).

In terms of research, enhancing PMTs’ lesson planning capacity through intervention requires careful consideration and flexibility, adapting to each context while remaining methodologically robust. The MaTeK intervention study aligned various data collection strategies to provide rich data and diverse perspectives on lesson planning, suggesting that it could serve as a blueprint for similar studies.

Regarding the study’s limitations and how these limitations can lead to further research, we acknowledge that the small number of participating PMT is a clear limitation. This could be addressed by including a larger number of participants from other pre-service mathematics education courses at different institutions. Additionally, only two textbook series from Norway were analysed, albeit the most commonly used ones. Analysing other textbook series might yield different results. Finally, the entire study was contextualized within Norway. Therefore, while the findings are meaningful, they may not be directly applicable to other countries or educational settings. Furthermore, findings from studies in other countries regarding lesson planning and suitable resources could contribute to a more detailed and comprehensive understanding of appropriate lesson planning activities for PMTs.

In conclusion, PMTs can develop lesson planning and design competence within initial teacher education when a thoughtful and scaffolded approach to lesson planning is employed. This is especially crucial during periods of curricular reform, when PMTs cannot rely to their past experiences as pupils in school or as students at university. We argue that PMTs require support in planning mathematics lessons by structuring their lesson plan to enhance, for example, their awareness of the curriculum (particularly with respect to R&P) and its changes under a reformed curriculum. Additionally, this support should help them focus on noticing pupil thinking; identifying potential learning trajectories in different mathematical areas; using curriculum resources in a considered way and effectively, and exploring innovative pedagogies (e.g., incorporating new technologies). Such support is best provided within communities of learning (whether in school or university) with peer and university teacher feedback. Lesson planning and the trial of lesson designs in the classroom should ideally go hand-in-hand, with ample time for critical reflection, particularly during times of curricular change. By framing curricular design as part of teachers’ career-long learning, this study contributes to the ongoing work of clarifying the developmental aspect of PMTs’ curricular capacities with the support of curriculum resources.

In terms of contribution, this study examines which resources were useful for PMTs’ lesson planning during a curriculum reform in Norway, focusing on both resources and practices. Lesson planning is viewed as a form of curriculum development, with textbooks and other resources playing a key role in translating curricular goals into actionable lesson plans and classroom practices. The research bridges the theoretical aspects of curriculum resources with their practical application, analysing how teachers interact with these tools to align their lessons with new curricular objectives.

## References

[CR1] Borge, I. C., Hole, A., & Grønmo, L. S. (2022). Mathematics education in Norwegian academic-track upper secondary school. In T. Rolfes, S. Rach, S. Ufer, & A. Heinze (Eds.), *Das Fach Mathematik in der gymnasialen Oberstufe [The subject of mathematics in the upper secondary school]* (pp. 157–175). Waxmann.

[CR2] Borko, H., Peressini, D., Romagnano, L., Knuth, E., Willis-Yorker, C., Wooley, C., Hovermill, J., & Masarik, K. (2000). Teacher education does matter: A situative view of learning to teach secondary mathematics. *Educational Psychologist*, *35*(3), 193–206. 10.1207/S15326985EP3503_5.

[CR3] Brown, M. W. (2009). The teacher-tool relationship: Theorizing the design and use of curriculum materials. In J. T. Remillard, B. A. Herbel-Eisenmann, & G. M. Lloyd (Eds.), *Mathematics teachers at work: Connecting curriculum materials and classroom instruction* (pp. 17–36). Routledge.

[CR4] Buchbinder, O., & McCrone, S. (2020). Preservice teachers learning to teach proof through classroom implementation: Successes and challenges. *The Journal of Mathematical Behaviour*, *58*, Article 100779. 10.1016/j.jmathb.2020.100779.

[CR5] Cevikbas, M., König, J., & Rothland, M. (2023). Empirical research on teacher competence in mathematics lesson planning: Recent developments. *ZDM – Mathematics Education*, *56*, 101–113. 10.1007/s11858-023-01487-2. 10.1007/s11858-023-01487-2 PMC10134687 37361445

[CR6] Chen, S., & Zhang, B. (2019). Improving prospective teachers’ lesson planning knowledge and skills through lesson study. In R. Huang, A. Takahashi, & J. P. da Ponte (Eds.), *Theory and practice of lesson study in mathematics. An international perspective* (pp. 549–575). Springer. 10.1007/978-3-030-04031-4_27.

[CR7] Ellis, A., Özgür, Z., & Reiten, L. (2019). Teacher moves for supporting student reasoning. *Mathematics Education Research Journal*, *31*, 107–132. 10.1007/s13394-018-0246-6.

[CR8] Fantilli, R., & McDougall, D. E. (2009). A study of novice teachers: Challenges and supports in the first years. *Teaching and Teacher Education*, *25*(6), 814–825. 10.1016/j.tate.2009.02.021.

[CR9] Goodlad, J. I. (Ed.) (1979). *Curriculum inquiry: The study of curriculum practice*. McGraw-Hill.

[CR10] Gueudet, G., Pepin, B., & Trouche, L. (Eds.) (2012). *From text to ‘lived’ resources: Mathematics curriculum materials and teacher development*. Springer. 10.1007/978-94-007-1966-8.

[CR11] Haggarty, L., & Pepin, B. (2002). An investigation of mathematics textbooks and their use in English, French and German classrooms: Who gets an opportunity to learn what? *British Educational Research Journal*, *28*(4), 567–590.

[CR12] Hanna, G., & Barbeau, E. (2008). Proofs as bearers of mathematical knowledge. *ZDM – Mathematics Education*, *40*(3), 345–353. 10.1007/s11858-008-0080-5.

[CR13] Hemmi, K., Lepik, M., & Viholainen, A. (2013). Analysing proof-related competences in Estonian, Finnish and Swedish mathematics curricula—towards a framework of developmental proof. *Journal of Curriculum Studies*, *45*(3), 354–378. 10.1080/00220272.2012.754055.

[CR14] Hiim, H., & Hippe, E. (2006). Praksisveiledning i lærerutdanningen. En didaktisk veiledningsstrategi. [Practice guidance in teacher training. A didactic guidance strategy.] Gyldendal akademisk.

[CR15] Hjardar, E., & Pedersen, J. E. (2020a). *Matematikk 8. Grunnbok*. [Mathematics 8. Main book] Cappelen Damm.

[CR16] Hjardar, E., & Pedersen, J. E. (2020b). *Matematikk 9. Grunnbok*. [Mathematics 9. Main book] Cappelen Damm.

[CR17] Jeannotte, D., & Kieran, C. (2017). A conceptual model of mathematical reasoning for school mathematics. *Educational Studies in Mathematics*, *96*, 1–16. 10.1007/s10649-017-9761-8.

[CR18] Kongsnes, A. L., & Wallace, A. K. (2020a). *Matemagisk 8*. Aschehaug.

[CR19] Kongsnes, A. L., & Wallace, A. K. (2020b). *Matemagisk 9*. Aschehaug.

[CR20] Lim, W., Son, J. W., & Kim, D. J. (2018). Understanding preservice teacher skills to construct lesson plans. *International Journal of Science and Mathematics Education*, *16*, 519–538. 10.1007/s10763-016-9783-1.

[CR21] NCTM (2009). Focus in high school mathematics: Reasoning and sense making. *National Council of Teachers of Mathematics*.

[CR22] Norwegian Directorate for Education and Training. (2019). *Curriculum for Mathematics year 1–10*. https://data.udir.no/kl06/v201906/laereplaner-lk20/MAT01-05.pdf?lang=eng.

[CR23] OECD (2003). The PISA 2003 assessment framework. Mathematics, reading, science and problem solving knowledge and skills. OECD Publications. 10.1787/9789264101739-en.

[CR24] Pepin, B. (2018). Enhancing teacher learning with curriculum resources. In L. Fan, L. Trouche, C. Qi, S. Rezat, & J. Visnovska (Eds.), *Research on mathematics textbooks and teachers’ resources. ICME 13 monographs* (pp. 359–374). Springer. 10.1007/978-3-319-73253-4_17.

[CR25] Pepin, B., & Gueudet, G. (2018). Curriculum resources and textbooks in mathematics education. In S. Lerman (Ed.), *Encyclopedia of mathematics education*, Springer. 10.1007/978-3-319-77487-9_40-7.

[CR26] Pepin, B., Gueudet, G., & Trouche, L. (2017). Refining teacher design capacity: Mathematics teachers’ interactions with digital curriculum resources. *ZDM – Mathematics Education*, *49*, 799–812. 10.1007/s11858-017-0870-8.

[CR27] Pepin, B., Gueudet, G., & Trouche, L. (2013). Investigating textbooks as crucial interfaces between culture, policy and teacher curricular practice: Two contrasted case studies in France and Norway. *ZDM – Mathematics Education*, *45*, 685–698. 10.1007/s11858-013-0526-2.

[CR28] Pepin, B., Gueudet, G., & Choppin, J. (2024). Transformation of mathematics education environments by digital resources. In B. Pepin, G. Gueudet, & J. Choppin (Eds.), *Handbook of digital resources in mathematics education*, Springer. 10.1007/978-3-030-95060-6_1-1.

[CR29] Radford, L. (2006). Algebraic thinking and the generalization of pattern: A semiotic perspective. In S. Alatorre, J. L. Cortina, M. Sáiz, & A. Méndez (Eds.), *Proceedings of the 28th annual meeting of the North American chapter of the international group for the psychology of mathematics education* (pp. 2–21). Universidad Pedagógica Nacional.

[CR30] Reid, D. A. (2022). ‘Reasoning’ in national curricula and standards. In J. Hodgen, E. Geraniou, G. Bolondi, & F. Ferretti (Eds.), *Proceedings of the twelfth Congress of the European society for research in mathematics education (CERME12)* (pp. 299–306). Free University of Bozen-Bolzano and ERME. https://hal.science/hal-03746833v2.

[CR31] Remillard, J. T. (2005). Examining key concepts in research on teachers’ use of mathematics curricula. *Review of Educational Research*, *75*(2), 211–246. 10.3102/00346543075002211.

[CR32] Rezat, S. (2012). Interactions of teachers’ and students’ use of mathematics textbooks. In G. Gueudet, B. Pepin, & L. Trouche (Eds.), *From text to ‘lived’ resources: Mathematics curriculum materials and teacher development* (pp. 231–246). Springer. 10.1007/978-94-007-1966-8_12.

[CR33] Rivera, F., & Becker, J. R. (2005). Figural and numerical modes of generalizing in algebra. *Mathematics Teaching in the Middle School*, *11*(4), 198–204. 10.5951/MTMS.11.4.0198.

[CR34] Sadak, M. (2021). How do pre-service mathematics teachers enhance their lessons through lesson study? *International Journal of Scholars in Education*, *4*(1), 15–25. 10.52134/ueader.896081.

[CR35] Schmidt, W. H., Houang, R. T., Sullivan, W. F., & Cogan, L. S. (2022). When practice meets policy in mathematics education: A 19 country/jurisdiction case study. 10.1787/07d0eb7d-en. OECD Education Working Papers, No. 268. OECD Publishing.

[CR36] Silverman, B., & Even, R. (2015). Textbook explanations: Modes of reasoning in 7th-grade Israeli mathematics textbooks. In K. Krainer & N. Vondrová (Eds.), *Proceedings of the ninth Congress of the European*. society for research in mathematics education (CERME 9) (pp. 205–212). Charles University in Prague, Faculty of Education and ERME. https://hal.science/hal-01281094v1.

[CR37] Slavíčková, M., Kohanová, I., Pepin, B., & Zatrochová, M. (2022). Enhancement of research excellence in mathematics teacher knowledge: Collaborative designing of lessons and learning progressions. In J. Hodgen, E. Geraniou, G. Bolondi, & F. Ferretti (Eds.), *Proceedings of the twelfth Congress of the European society for research in mathematics education (CERME12)* (pp. 3698–3699). Free University of Bozen-Bolzano and ERME. https://hal.science/hal-03749499v1.

[CR38] Stephan, M. (2020). Socio-mathematical norms in mathematics education. In S. Lerman (Ed.), *Encyclopedia of mathematics education* (pp. 802–805). Springer. 10.1007/978-3-030-15789-0_143.

[CR39] Stylianides, G. J., & Stylianides, A. J. (2017). Research-based interventions in the area of proof: The past, the present, and the future. *Educational Studies in Mathematics*, *96*, 119–127. 10.1007/s10649-017-9782-3.

[CR40] Stylianides, A. J., & Stylianides, G. J. (2022). Introducing students and prospective teachers to the notion of proof in mathematics. *The Journal of Mathematical Behavior*, *66*, Article 100957. 10.1016/j.jmathb.2022.100957.

[CR41] Thijs, A., & van den Akker, J. (2009). *Curriculum in development*. Netherlands Institute for Curriculum Development (SLO).

[CR42] Valenta, A., & Enge, O. (2020). Bevisrelaterte kompetanser i læreplanen LK20 for matematikk i grunnskolen. [Proof-related competences in the LK20 curriculum for mathematics in primary school.]. *Acta Didactica Norden*, *14*(3). 10.5617/adno.8195.

[CR43] Valverde, G. A., Bianchi, L. J., Wolfe, R. G., Schmidt, W. H., & Houng, R. T. (2002). *According to the book: Using TIMSS to investigate the translation of policy into practice through the world of textbooks*. Kluwer Academic. 10.1007/978-94-007-0844-0.

[CR44] van den Akker, J. (2003). Curriculum perspectives: An introduction. In J. van den Akker, W. Kuiper, & U. Hameyer (Eds.), *Curriculum landscapes and trends* (pp. 1–10). Springer. 10.1007/978-94-017-1205-7_1.

[CR45] Weingarden, M., & Buchbinder, O. (2023). Teacher learning to teach mathematics via reasoning and proving: A discursive analysis of lesson plans modifications. *Frontiers in Education*, *8*, Article 1154531. 10.3389/feduc.2023.1154531.

